# Recent Updates on DTD (D-Tyr-tRNA^Tyr^ Deacylase): An Enzyme Essential for Fidelity and Quality of Protein Synthesis

**DOI:** 10.3389/fcell.2016.00032

**Published:** 2016-04-26

**Authors:** Tarun K. Bhatt, Rani Soni, Drista Sharma

**Affiliations:** Department of Biotechnology, School of Life Sciences, Central University of RajasthanBandar sindri, India

**Keywords:** DTD, D-amino acids, 3D structure of DTD, Enzyme mechanism od DTD, Protein synthesis checkpoints

## Abstract

During protein synthesis, there are several checkpoints in the cell to ensure that the information encoded within genetic material is decoded correctly. Charging of tRNA with its cognate amino acid is one of the important steps in protein synthesis and is carried out by aminoacyl-tRNA synthetase (aaRS) with great accuracy. However, due to presence of D-amino acids in the cell, sometimes aaRS charges tRNA with D-amino acids resulting in the hampering of protein translational process, which is lethal to the cell. Every species has some mechanism in order to prevent the formation of D-amino acid-tRNA complex, for instance DTD (D-Tyr-tRNA deacylase) is an enzyme responsible for the cleavage of ester bond formed between D-amino acid and tRNA leading to error free translation process. In this review, structure, function, and enzymatic mechanism of DTD are discussed. The role of DTD as a drug target is also considered.

## Introduction

Protein synthesis process results in the production of functional proteins and the accuracy of this process determines the fate of the cell. There are several modes by which cell corroborates the fidelity of the translation process (Jonak et al., 1980; Bhuta et al., 1981; Alksne et al., 1993; Liu and Liebman, 1996; Lin et al., 2010). Both D and L amino acids are present in cell (Broccardo et al., 1981; Dunlop et al., 1986; Hashimoto et al., 1995; Wolosker et al., 1999; Nagata et al., 2006). The discrimination between D and L amino acids and incorporation of only L-amino acids is the decisive checkpoint (Yamane et al., 1981; Ibba and Söll, 1999). The aminoacyl-tRNA synthetase (aaRS) is an enzyme responsible for charging of tRNA with L-amino acids (Ibba et al., 2000; Rodnina and Wintermeyer, 2001; Francklyn et al., 2002). However, in some cases, aaRS may tag tRNA with D-amino acids (Giegé et al., 1998; Yadavalli and Ibba, 2012) resulting into the formation of D-aa-tRNA complex. The resulting complex engages the tRNA pool present in the cell due to which L-amino acids are destitute of their cognate tRNA partners. The complex is very lethal and in order to rescue cell, formed D-aa-tRNA complex must be cleaved to release tRNA molecules (Soutourina et al., 1999, 2004) and it is done by the proofreading activity of D-Tyr-tRNA deacylase (DTD) responsible for hydrolyzing D-aa-tRNA complex. It causes the cleavage of bond formed between D-amino acids and tRNA thus making tRNA available to coalesce with L-amino acids (Wydau et al., 2009; Zheng et al., 2009). It was first reported in *Escherichia coli* and *Bacillus subtilis* that tRNA molecules are charged with D-tyrosine in the presence of Tyrosyl-tRNA synthetase (Owens and Bell, 1968). Later, a report of D-Valine, D-Aspartate, and D-Tryptophan coupling with their respective tRNA was published by different scientific groups (Soutourina et al., 2000). However, the first report of an enzyme capable of hydrolyzing ester bond of D-Tyr-tRNA was published by Calender and Berg (Calendar and Berg, 1967). A study showed the existence of DTD in all the three domains of life, including human, archaebacteria, and malaria parasite (Bhatt et al., 2010). To date, three types of DTD are known with similar functions with subtle changes in sequences (Wydau et al., 2009). In humans, two types of DTD proteins are present viz DTD1, DTD2. DTD1, part of DUE-B protein has been biochemically and structurally characterized for its deacylase function (Kemp et al., 2007). It would be interesting to determine deacylase activity and structural architect of DTD2 protein in comparison to DTD1. The active site motif of DTD2 is “PQAT” in lieu of “SQFT.” It was put forward that in *E. coli*, the deletion of DTD gene led to higher toxicity of D-amino acids conferring the role of DTD in combating the ill effects of D-chirality (Soutourina et al., 2004). The presence of DTD in all forms of life and its role in striving against the D-amino acid toxicity makes it an essential gene to be retained for survival. In this review, we will be focusing on the structure and function of DTD enzyme known so far. The possibility of DTD as a drug target will also be accentuated.

## Structure of DTD

Biochemical studies had established the presence of an editing enzyme for D-amino acids, but lacked any structural evidence related to it. The information on crystal structure and three dimensional coordinates of *E. Coli* DTD enzyme was proposed by Ferri-Fioni et al. (2001). It was indicated that the asymmetric unit consists of protein dimer, akin to the results obtained from gel filtration chromatography techniques. The overall structure of DTD belongs to α/β class of protein, containing five-stranded mixed β sheet, three-stranded anti-parallel β sheet covered by two parallel α helices (Ferri-Fioni et al., 2001). Due to conservedness of the function of gene among different species, it was considered that the structural architecture of protein is also similar. Different studies were conducted in order to resolve the crystallographic structure of the enzyme from different sources such as *Haemophilus influenza, Aquifex aeolicus, Homo sapiens, Leishmania major*, and *Plasmodium falciparum* (Lim et al., 2003; Kemp et al., 2007; Bhatt et al., 2010). The structural similarity of DTD enzyme among different species was further reestablished by Dali score of 17–24 and root mean square deviation of 1.1 to 1.9 A° (Bhatt et al., 2010). The probable active site cavity consisting of “SQFT” motif was also found to be well conserved amidst all structures (Bhatt et al., 2010). In addition to stand-alone DTD proteins, the DTD-like domain (Pab-NTD) was also found appended to threonyl-tRNA synthetase (ThrRS) of *Pyrococcus abyssi* with the similar hydrolytic activity of DTD (Hussain et al., 2006; Ahmad et al., 2015). The presence of a free standing DTD probably would have evolved from ThrRS under extensive pressure of D-amino acid toxicity. However, further studies are required to authenticate and explore the evolution of free standing DTD from appended ThrRS and vice-versa. The conserved structure and function of DTD in almost all the known species lays emphasis upon the importance of having such proofreading enzyme for hassle-free translation.

## Enzymatic mechanism of DTD

The catalytic motif “SQFT” (Serine, Glutamine, Phenylalanine, and Threonine) responsible for the function of protein is highly conserved amongst all domains of the life. It is a small amino acid motif where “Threonine” is highly conserved in all DTD, being the main catalytic residue whereas in case of human DTD2 protein, “Serine” is replaced by “Proline” and “Phenylalanine” is replaced by “Alanine.” The proposed active site of the DTD is formed at the dimer interface with “SQFT” motif of first monomer and “nNXG(V/F)T” motif of the second monomer, as supported by the three-dimensional structure of *E. coli* DTD (Ferri-Fioni et al., 2001). The interaction of D-Tyr-tRNA with the active site of DTD from *H. Influenza* gives an insight about the probable mechanism of action. A study had elucidated that the DTD hydrolysis involves general base mechanism, where “Threonine” act as a nucleophile and attack on the carbonyl group of D-Tyr linked to the terminal adenine of tRNA via an ester bond (Lim et al., 2003). However, the experimental evidence for the proposed mechanism was provided by high-resolution enzyme–substrate structures solved using x-ray crystallographic studies (Bhatt et al., 2010; Yogavel et al., 2010). The structural snapshots of the *Plasmodium* DTD with different substrate like molecules nicely decipher the mechanism of action by proposing three sub-sites viz. transition site, active site and exit site. Study conducted by Lim demonstrated that the O atom present in side chain of threonine residue functions as the main nucleophile and phenylalanine and glutamine have role in stabilization of oxyanion hole resulting into the cleavage of the ester bond formed between D-amino acids and tRNA (Lim et al., 2003). The mutational study involving the replacement of “Threonine” with “Alanine” also substantiated the role of threonine as the main active site residue (Bhatt et al., 2010). Although, a complex structure of DTD and charged-tRNA molecule would have authenticated the proposed enzyme mechanism which is presently based on a substrate like molecules such as free amino acids and nucleotides. These findings concluded the role of threonine as an active site residue enabling the action of the DTD on D-amino acids containing tRNA molecule. However, the mechanism of discrimination between D and L amino acids by DTD enzyme is yet to be elucidated. The enantio-selectivity of the DTD explained by the presence of Gly-Cys-Pro dipeptide in PfDTD which is responsible for maintaining homochirality by selecting only D-amino acids and rejecting L-amino acids (Ahmad et al., 2015). Although, most of the studies related to the enzymatic mechanism have been conducted for *Plasmodium* DTD, but it is assumed that it will be valid for all DTD proteins owing to highly conserved sequences.

## DTD as drug target

Highly conserved nature of DTD makes it less attractive target for drug development. However, DTD may be targeted as a potential drug target if D-amino acids are used in combination with known inhibitors. For example, when an inhibitor (N, N-bis[4-amino-2-methyl-6-quinolinyl]urea) was used for *in-vitro* malaria parasite inhibition assays, it showed inhibition of parasite growth at micromolar concentration. But when used in combination with D-amino acids, better inhibitory activity on parasite growth was observed. The underlying mechanism of inhibition by quinoline derivative involves the conversion of free ferriprotoporphyrin into crystalline hemozoin as well as inhibition of DTD activity leading to detrimental effect on parasite growth (Pagola et al., 2000; Bhatt et al., 2010). Such combination of D-amino acids and drug-like molecules could lead to possible inhibition of pathogen by targeting DTD protein. In addition, if subtle differences between structure of human DTD and its homologs in other species are found, it might be utilized for developing drug like molecules specific to non-human DTD.

**Figure 1:**
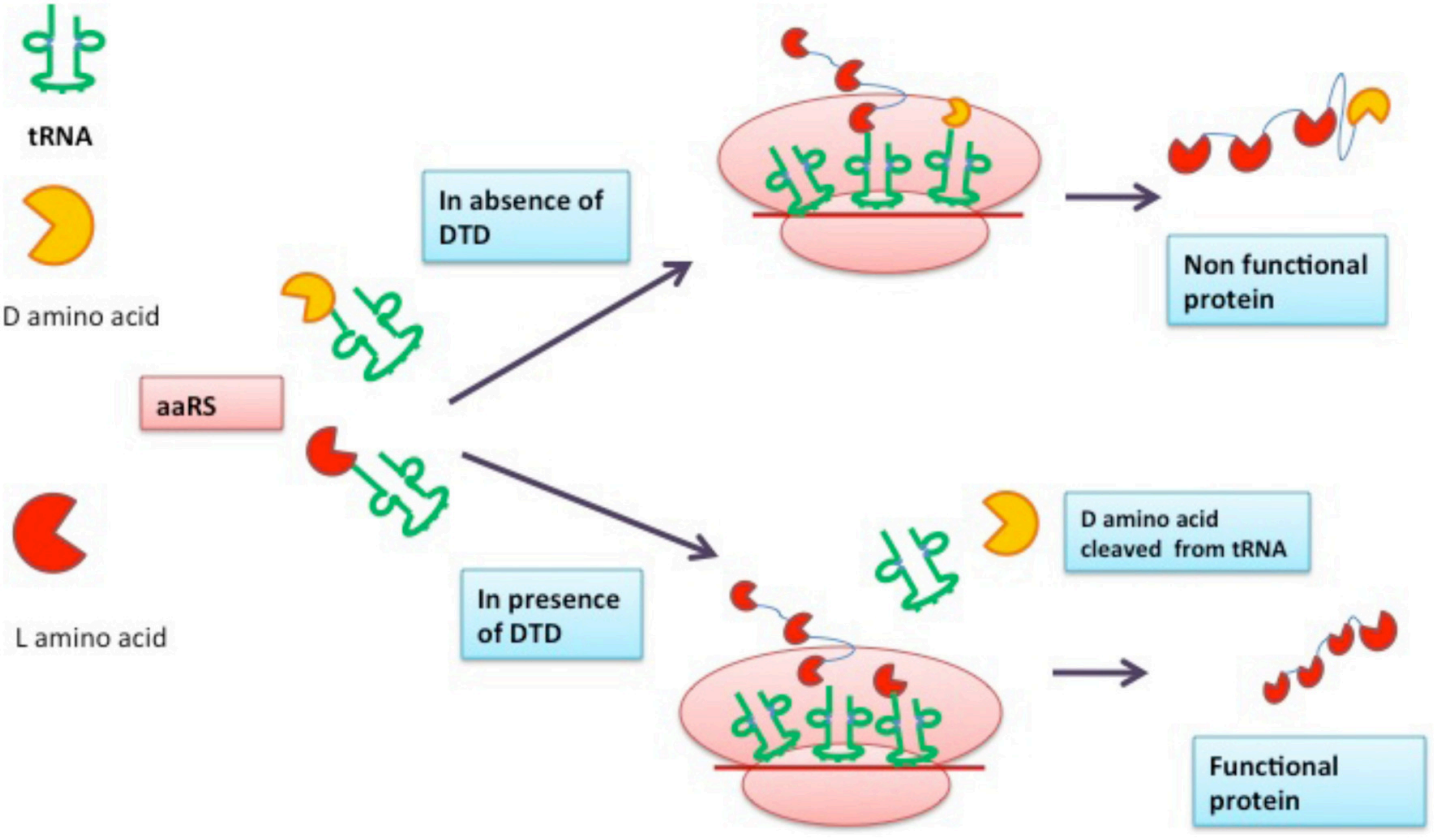
**Role of DTD in fidelity and quality of protein synthesis**.

## Conclusion

The presence of both forms of chiral amino acids (D and L) in living beings has been known from long, but the rationale behind the selection of L-form of amino acids is still under cloud. However, the discovery of DTD enzyme has answered many important questions covering how translational machinery ensures the participation of only L-amino acids in growing chain, the specificity of DTD toward D-amino acids etc. The large quantity of biochemical and structural data have decoded the enzymatic mechanism of DTD and stabilized “SQFT” and “Gly-Cys-Pro” motifs for their role in catalysis and selectivity respectively. Interestingly, being a crucial enzyme in maintaining fidelity of translation machinery in terms of stereo-specific amino acid selection, modification of DTD may be utilized in the development of an *in-vivo* system for generation of peptides/proteins containing D-amino acids. Subtle changes in residues responsible for the enantio-selectivity could alter the property of DTD in a way suitable for entry of D-amino acids into growing chain. This may lead to production of D-amino acids containing peptides/proteins, known for various therapeutic uses (Kolodkin-Gal et al., 2010). There is a huge window of opportunity in this direction of production of D-amino acid peptides using *in-vivo* system of DTD, yet to be explored. In addition, atomic level information on DTD structure and catalysis might pave the way for designing specific inhibitors in combination with D-amino acids against microbes and parasites.

## Author contributions

TKB has written the paper. RS and DS provided the data.

## Funding

The author is thankful to UGC, StartUp Scheme, Govt. of India for providing financial assistance.

### Conflict of interest statement

The authors declare that the research was conducted in the absence of any commercial or financial relationships that could be construed as a potential conflict of interest.
